# Emerging issues in genomic selection

**DOI:** 10.1093/jas/skab092

**Published:** 2021-03-27

**Authors:** Ignacy Misztal, Ignacio Aguilar, Daniela Lourenco, Li Ma, Juan Pedro Steibel, Miguel Toro

**Affiliations:** 1 Department of Animal and Dairy Science, University of Georgia, Athens, GA 30602, USA; 2 Instituto Nacional de Investigación Agropecuaria (INIA), 90200 Canelones, Uruguay; 3 Department of Animal and Avian Sciences, University of Maryland, College Park, MD 20742, USA; 4 Department of Animal Science, Michigan State University, East Lansing, MI 48824, USA; 5 Departamento de Producción Agraria, Universidad Politécnica de Madrid, Madrid, Spain

**Keywords:** genomic evaluation, genomic selection, genomwide association studies, large data, stability of genomic predictions

## Abstract

Genomic selection (**GS**) is now practiced successfully across many species. However, many questions remain, such as long-term effects, estimations of genomic parameters, robustness of genome-wide association study (**GWAS**) with small and large datasets, and stability of genomic predictions. This study summarizes presentations from the authors at the 2020 American Society of Animal Science (**ASAS**) symposium. The focus of many studies until now is on linkage disequilibrium between two loci. Ignoring higher-level equilibrium may lead to phantom dominance and epistasis. The Bulmer effect leads to a reduction of the additive variance; however, the selection for increased recombination rate can release anew genetic variance. With genomic information, estimates of genetic parameters may be biased by genomic preselection, but costs of estimation can increase drastically due to the dense form of the genomic information. To make the computation of estimates feasible, genotypes could be retained only for the most important animals, and methods of estimation should use algorithms that can recognize dense blocks in sparse matrices. GWASs using small genomic datasets frequently find many marker-trait associations, whereas studies using much bigger datasets find only a few. Most of the current tools use very simple models for GWAS, possibly causing artifacts. These models are adequate for large datasets where pseudo-phenotypes such as deregressed proofs indirectly account for important effects for traits of interest. Artifacts arising in GWAS with small datasets can be minimized by using data from all animals (whether genotyped or not), realistic models, and methods that account for population structure. Recent developments permit the computation of *P*-values from genomic best linear unbiased prediction (**GBLUP**), where models can be arbitrarily complex but restricted to genotyped animals only, and single-step GBLUP that also uses phenotypes from ungenotyped animals. Stability was an important part of nongenomic evaluations, where genetic predictions were stable in the absence of new data even with low prediction accuracies. Unfortunately, genomic evaluations for such animals change because all animals with genotypes are connected. A top-ranked animal can easily drop in the next evaluation, causing a crisis of confidence in genomic evaluations. While correlations between consecutive genomic evaluations are high, outliers can have differences as high as 1 SD. A solution to fluctuating genomic evaluations is to base selection decisions on groups of animals. Although many issues in GS have been solved, many new issues that require additional research continue to surface.

## Introduction

Genomic selection (**GS**) is now implemented in all major farm animal species. Its main purpose is accelerating genetic trends by increasing the accuracy of selection, particularly for young animals, and reducing generation interval. The increase in selection accuracy may be particularly important for low heritability traits where conventional selection is slow. Many of the reports indicate that genetic trends under GS have indeed accelerated.

The advantages of GS may diminish in the long run. Intensive selection leads to a reduction of the additive genetic variance because of the Bulmer effect, with a resulting impact on genetic gain according to the breeders’ equation (Van Grevenhof et al., 2012). Because gene frequencies can change faster for genes associated with higher heritability traits, pleiotropy may intensify antagonistic genetic correlations (Georges et al., 2019). Epistatic modifications (Mackay, 2014; Ma et al., 2019) that were slow prior to GS may accelerate under GS, reducing the utility of old data. Some studies suggested that some of these phenomena have already occurred. VanRaden et al. (2014) reported that realized accuracies in Holstein are higher when assuming lower heritabilities. Hidalgo et al. (2020) found that the heritability for some traits in pigs was halved, and genetic correlations between production and fertility traits became more extreme.

Finding whether genetic parameters have changed over time requires estimation of these parameters generation by generation. Parameter estimation methods before GS such as restricted maximum likelihood (**REML**) and Bayesian via Gibbs sampling could analyze large datasets because the mixed model equations were sparse (Misztal, 2008). Conversely, mixed model matrices are dense under GS; thus, computations are more complex. According to Henderson (1984), estimation is unbiased if all data used for selection are included in best linear unbiased prediction (**BLUP**). If unbiased estimation under GS requires all genomic, pedigree, and phenotypic data used for selection to account for genomic preselection (Patry and Ducrocq, 2011; Masuda et al., 2018), then computations for large commercial datasets will be daunting.

Many of the studies use small genomic datasets for a genome-wide association study (**GWAS**). They frequently find many associations (Galliou et al., 2020, Leal-Gutiérrez et al., 2020), whereas studies using much bigger datasets find only a few (e.g., Jiang et al., 2019b). Assuming that studies with large datasets are more trustworthy, then many associations from small studies must be spurious. Possible reasons for spurious signals include data/population structure, and in particular, omission or simplified treatment of ungenotyped data, simplified analytical models, and problems with computing *P*-values in more complex analyses. For more reliable GWAS, all pertinent data (whether genotyped or not) need to be used, realistic models should be applied, and methods should account for population structure.

Genetic predictions with BLUP before GS were stable even when accuracies were low, except when animals acquired new phenotypes or new daughters with phenotypes. The industry was pleased with the stability of BLUP. However, genomic predictions are less stable; a top-ranked animal can easily drop in the next evaluation. Thus, the industry has a crisis of confidence in genomic evaluations. Genomic evaluations change because all animals with genotypes are connected. In particular, new phenotypic data for one animal cause small but correlated changes in all animals. Additionally, model modifications such as blending level of genomic relationships with pedigree relationships and scaling of the genomic relationships cause further changes. While correlations between consecutive genomic evaluations are high, outliers can have substantially bigger differences. The question is whether fluctuations in genomic estimated breeding values (**GEBV**s) are harmful to the industry or they are just an integral part of GS.

The objectives of this study were: 1) to examine theoretical issues affecting genetic selection, 2) to discuss strategies for parameter estimation under GS, 3) to look at GWAS from the perspective of large datasets, 4) to look at GWAS from the perspective of small datasets when the use of all available information is crucial to eliminate spurious responses, and 5) to discuss degrees of fluctuation of GEBV and ways the industry can manage these fluctuations.

## Theoretical Issues Affecting Genetic Selection

### Higher-order linkage disequilibrium

Linkage disequilibrium (**LD**) is an important phenomenon relevant to both GWAS and GS. The focus of research until now has been on LD between two loci.

It has been overlooked that for three loci (A, B, and C) a third-order LD coefficient could appear :

$$\begin{array}{l} D_{ABC}=p_{ABC}-\;p_{A}D_{BC}-\;p_{B}D_{AC}-\;p_{C}D_{AB}-\;p_{A}p_{B}p_{C} \end{array}$$ (1)

where *p*_*ABC*_ is the frequency of the ABC triplet; *p*_*A*_, *p*_*B*_, and *p*_*C*_ are the gene frequencies; and *D*_*AB*_, *D*_*BC*_, and *D*_*AC*_ are the pairwise disequilibrium coefficients. The third-order LD can be interpreted as nonindependence among single-nucleotide polymorphism (**SNP**) triplets unaccounted for by pairwise coefficients. The maximum third-order LD occurs when the three loci are at intermediate allele frequencies and second-order disequilibria are zero and range between −1/8 and 1/8.

There are few estimates of third-order LD in livestock species. Gomez-Raya et al. (2018) found third-order LD to be common in a composite line of Iberian pigs based on 26,347 SNPs from 306 sows, probably due to admixture.

### Phantom genetic parameters

Third-order LD is relevant in the context of spurious values of genetic parameters when using SNP markers, such as apparent dominance or epistatic variances when they do not exist. Consider three loci and the existence of third-order LD *only*. The first model consists of two quantitative trait loci (**QTL**s; Q/q and R/r) and one marker (M/m); if there is epistatic variance in the QTL, then a dominance variance would appear in the marker. The second model includes one QTL (Q/q) and two markers (M/m and N/n). If the QTL is additive, only an apparent additive by additive (**A × A**) variance would be suggested by the markers, and if the QTL is dominant, only an apparent A × A and an apparent dominance by dominance (D×D) variance would appear in the markers.

Imperfect LD can generate the illusion of epistasis even when the underlying genetic architecture is purely additive. de los Campos et al. (2019) showed that phantom epistasis could be a very serious problem in GWAS (with rejection rates against the additive model greater than 0.28 for nominal *P*-values of 0.05, even when the model is purely additive) and demonstrated that the magnitude of the problem was even greater with large sample sizes.

### Distinction between inferential and prediction problems

Phantom dominance or epistasis creates inferential problems limiting the possibility to learn about causal effects because either epistasis or dominance is detected when they do not exist. However, models that do not accurately represent the genetic architecture of a trait could be useful for improving prediction models. In 10,000 simulations of random values of gametic frequencies and genetic effects, we found that in 0.5% of cases V′ _Amark_ > V_Aqtl_ (one QTL and two markers) and in 1.7% of cases V′ _Amark_ > V_Aqtls_ (two QTLs and one marker), where V′ _A_ is the apparent variance explained by the markers and V_A_ the true additive variance, respectively. Recently, Schrauf et al. (2020) in a simulation study inspired by publicly available *Arabidopsis* and rice datasets illustrated that, due to phantom epistasis, epistatic models may also predict the genetic value of an underlying purely additive genetic architecture better than additive models, when the marker density is low.

### Genetic vs. genomic correlation

In an additive context, we could expect a missing heritability for one trait, but could we also expect a missing genetic correlation between two traits? This is a difficult question, and, according to Gianola et al. (2015), genomic correlation could be greater than, lower than, or even of opposite sign to the true genetic correlation. Gianola et al. (2015) studied several cases of a simple model with two QTLs and two markers. In case 1, there was no pleiotropy, and two QTLs were in linkage equilibrium (**LE**), thus the genetic correlation was zero; but because of LD between markers, there was a nonzero genomic correlation. In case 2, the only source of genetic correlation between traits was LD between QTLs, which could be completely lost in a genomic analysis. In case 3, two QTLs affected both traits (pleiotropy), and genetic and genomic correlations differed in magnitude and even in sign depending on the pattern of LD. In conclusion, speculating about genetic correlations, and even about their causes (e.g., pleiotropy), using genomic data is often conjectural.

### Changes in genetic parameters with selection

#### Changes in environmental variance

The search for more uniform animal performance has increased its economic importance in livestock. Genetic control of environmental variance offers opportunities to increase this uniformity. Classical models normally assume that the environmental or residual variance is constant. However, there is evidence that environmental variance can also be under genetic control with a heritability of about 0.10. This has been shown in pigs (birth weight, stillbirths, and litter size), sheep (litter size), poultry and beef cattle (weight), and dairy cattle (milk yield). Therefore, it is possible to decrease environmental variance by selection, but large phenotypic and genomic datasets are needed (see the recent review of Iung et al., 2020).

#### Changes in genetic variances with inbreeding

The implementation of GS has increased rates of inbreeding mainly due to higher prediction accuracies and shorter generation intervals. It is important to estimate EBVs and inbreeding using the same source of information (i.e., either based on pedigree or genomic information). Thus, the rate of inbreeding per generation in Holstein increased by 1.4% in the last 10 yr. A higher rate of inbreeding could compromise fitness traits and long-term genetic gains. There are methods to manage inbreeding in a GS program. Genomic information can be utilized to identify and eliminate lethal recessives and to perform optimal contributions and optimal mate allocations.

#### Changes in genetic variances due to the Bulmer effect

It is well known that directional selection creates negative LD (i.e., + − + −). This is not obvious because ++++ chromosomes are the most favored, and thus one might expect positive LD. However, in the simplest demonstration, Maynard Smith (1998) considers a haploid model ab, aB, Ab, and AB with equal frequencies of 0.25. Phenotypic values are 0, 1, 1, and 2, and phenotype fitness values are (1 − s), 1, 1, and (1 + s), where s is the selection coefficient. Before selection, the LD is 0.25 × 0.25 − 0.25 × 0.25 = 0, and, after selection, LD will be 0.25 (1 − s) × 0.25 (1 + s) − 0.25 × 0.25 = −s^2^/16.

Reductions in selection response with BLUP due to the Bulmer effect are independent of heritability and depend only on selection intensity. Their values have ranged from 0.22 to 0.27. Reduction in selection response is greater with BLUP than with phenotypic selection. However, the selection response is still larger with BLUP than with phenotypic selection.

The Bulmer effect has been shown: 1) in old experiments (1970s) with *Drosophila* where suppression of crossing-over reduced the long-term phenotypic selection response, especially for small effective population size; 2) in many simulation results; and 3) in analyses of empirical data. For example, in Manech Tête Rousse dairy sheep (1,842,295 records of milk yield and 530,572 females), the loss of genetic variance was about 13% for females. The Bulmer effect had more impact (10%) than the buildup of coancestry (3%) in 30 yr (Macedo et al., 2021). 

Van Grevenhof et al. (2012) established that the reduction in response due to the Bulmer effect was the same for GS as for selection based on traditional BLUP, and it was independent of the accuracy of selection. However, it seems that intensive changes caused by GS can further reduce the genetic variation and strengthen undesirable genetic correlations (Hidalgo et al., 2020).

A positive aspect of GS is that we can act on the within-family variance (Mendelian sampling variance). Thus, we can better use the Mendelian sampling variance by selecting parents on an index composed of GEBV and gametic variabilities of selection candidates. According to Bijma et al. (2020), GS can increase the probability of breeding a top-ranking individual by 36% and response to selection by 3.6% in the Holstein–Friesian dairy cattle population. In addition, optimal mating partners that maximize the variance of the offspring can be identified.

### Selection and recombination

The other side of the coin of the Bulmer effect is that if we increase recombination rate, we can enhance selection efficiency. A higher recombination rate would reduce LD between causative variants and release genetic variance.

Now the question is if there is genetic variance for the recombination rate. The answer is positive as shown in various lines of evidence (reviewed by Battagin et al., 2016): 1) old experiments with *Drosophila* and *Tribolium* where selection succeeded in both increasing and decreasing recombination rates; 2) domesticated mammals had higher chiasma frequencies than wild mammals; 3) heritability estimates of recombination rates were substantial (0.12 to 0.22 in sheep, 0.22 to 0.26 in cattle, 0.05 to 0.07 in pigs, 0.16 to 0.17 in chicken, and 0.30 in humans); and 4) QTLs and genes affecting recombination rate have been detected either for general and for specific regions. For example, Ring Finger Protein 212 acts as a regulator for crossing-over during meiosis. Lastly, targeted recombination is taken seriously by plant breeders.

Although in principle an increase in the rate of recombination can enhance the efficiency of selection, it can also break down favorable combinations. For example, the contribution of A × A variance to selection response depends on the rate of recombination. Thus, the selection response is equal to:

$$\begin{array}{l} R_{t}=i\sigma_{P}(th^{2}+\frac{V_{AA}}{V_{P}}) \end{array}$$ (2)

with free recombination, and:

$$\begin{array}{l} R_{t}=i\sigma_{P}(th^{2}+t\frac{V_{AA}}{2V_{P}}) \end{array}$$ (3)

with no recombination, where $R_{t}$ = response to selection at generation t, i = intensity of selection, $\sigma_{P}$ = phenotypic standard deviation, $h^{2}$ = heritability, $V_{AA}$ = A × A genetic variance, and $V_{P}$ = phenotypic variance. In the context of GS, the situation is more complex because recombination could lower the accuracy of GS because it erodes LD between markers and QTLs unless causal genes are included in the SNP panel.

### Genetic architecture of complex traits

How do we expect the genome to be organized as a consequence of the joint action of pleiotropy, epistasis, recombination, and selection? There are two main proposals: 1) a modular model (restricted pleiotropy model; Wagner and Altenberg, 1996) consisting of groups of linked elements coding for the same functional trait that can evolve more or less independently of other modules coding for a different functional trait, in such a way that the behavior of elements inside a module depends little on factors external to the module; 2) an omnigenic model (Liu et al., 2019) that has *core* genes that affect the trait directly and *peripheral* genes that affect core genes through regulatory effects in such a way that almost every gene affects many characters (universal pleiotropy) and almost every character is aﬀected by many genes. Although the core genes are the relevant genes, most of the heritability is determined by variation in peripheral genes. The last model will impose a higher cost on multitrait selection.

## Parameter Estimation Under GS

Genetic parameter estimation was well established before the genomic revolution. It involved choosing a complete dataset or a subset if the complete dataset was too large, then using one of the two well-established methodologies resistant to selection biases, such as REML or Bayesian via Gibbs sampling (see Misztal (2008) for a review). Both methodologies relied on the mixed model equations being sparse and, therefore, easy to store even for millions of equations, and REML computations were facilitated by efficient sparse matrix inversion procedures (Misztal, 2008). REML methodology would support datasets with up to a million animals, with cubic increases in computations by trait. General REML becomes unstable with many traits. Bayesian methodologies offered lower memory requirements but the running time in terms of the number of samples had to be determined experimentally. Increase in computations with multiple traits could be linear.

### Changes due to genomic information

When genomic information is available, parameters could be obtained using SNP-only models with software such as GS3 (Legarra et al., 2011). In this case, either only phenotypes of genotyped animals are considered or genotyped animals use pseudo-phenotypes such as deregressed proofs, with corresponding problems (Legarra et al., 2014). Therefore, the focus of this section is limited to single-step genomic BLUP (**ssGBLUP**), which can accommodate both genotyped and ungenotyped animals directly.

The use of genomic information in REML using ssGBLUP (**ssGREML**) leads to dense equation systems. The inverse of the pedigree relationship is usually constructed using Henderson’s rules (Henderson, 1984), with at most nine nonzero elements per animal. The inverse of the relationship matrix H in ssGREML combines pedigree and genomic relationships and contains a genomic relationship matrix G. Matrix G is full, and thus it has a strong effect on the sparsity of the mixed model equations and subsequently on computing costs. Consider a single-trait model with two effects, 1 million animals with phenotypes and a nearly complete pedigree. The number of nonzero elements in the mixed model equations would be approximately 5 million, a number easily stored on a current computer. With added genomic information from 3,000 animals, the number of nonzero elements in the mixed model equations will double to about 10 million; hence, they will no longer be sparse. With 50,000 genotyped animals available at many breeding organizations, the number of nonzero elements in the mixed model equations will be over 1 billion. However, regular sparse matrix techniques are inefficient for matrices with dense blocks. Thus, Masuda et al. (2014, 2015) developed a sparse matrix package that identifies dense blocks and processes these blocks efficiently (up to 100 times faster than previous algorithms). When included in ssGREML, a four-trait analysis of a model with 200,000 animals, including 15,000 genotyped animals, would finish in about 10 h. Increases in computations would be cubic with the number of traits and the number of genotyped animals.

### Data selection and parameter estimation

High computing times with genomic information raises the question of whether parameter estimation should exclude genomic information, especially when computations are expensive. When genomic information is not used for selection, the only benefit from genomic analyses would be reduced SE of estimates (Forni et al., 2011). When genomic information is used for selection, the expectation of the Mendelian sampling is no longer 0, and nongenomic analyses are biased by preselection (Patry and Ducrocq, 2011; Masuda et al., 2018). The extent of biases will depend on the intensity of selection, the type of genotyping, and the selection of datasets for analyses. Cesarani et al. (2019) simulated populations with and without selection, with random and selective genotyping, and with few and many generations of data. Analyses included ignoring genetic information (REML), ignoring genotypes from ungenotyped animals (**GREML**), and using all information (ssGREML). Biases with unselected populations were small especially with more than one generation of data. Biases were particularly strong for GREML when the best animals were genotyped. ssGREML was relatively unbiased but it had the highest cost.

Another study (Wang et al., 2020) looked at the impact of the proportion of selective genotyping on biases. With selective genotyping, the additive genetic variance was inflated up to six times, and the phenotypic variance that is usually very stable across models almost doubled in some analyses. It is unclear whether the above results are valid or are artifacts of details of the implementation of ssREML. In general, estimates by ssGREML are affected by compatibility between pedigree and genomic relationships as well as by effective quality control. One test for the compatibility is estimating variances with simulated data without selection. In such a case, the estimates should be identical for REML and ssGREML, regardless of the strategy for genotyping.

### Changes in parameters over time

One consequence of GS could be faster changes of genetic parameters. In particular, if the additive genetic variance is decreasing and unfavorable genetic correlations are increasing, expected genetic gains based on prior parameters will not be realized. However, estimating parameter changes is difficult. Under an additive model, when all data used for selection are included in the estimation, the estimated parameters correspond to those of the base population. Therefore, if the base population does not change, the estimated parameters will be the same even if the parameters for the youngest generations change.

Changes over time can be estimated either with a random regression model (**RRM**) over time or using data slices (Tsuruta et al., 2004). With an RRM over time, parameters can be functions of time. However, the shape of the function depends on functions used in RRMs. A linear function will restrict changes to quadratic for variances and linear for covariances, whereas with a quadratic function, changes will be the fourth degree for variances and quadratic for covariances. Using a higher degree polynomial improves the ability of the RRM to describe more complex changes, but at a cost of more parameters, possibly with insufficient data to properly estimate those parameters. Another problem with the use of RRM with genomics is high cost, increasing with higher-order polynomials. With data slices, computations involve only a fraction of the data, greatly decreasing computations. However, the size of the data slice creates a tradeoff between modeling fast changes and biases from using truncated data.

A good illustration of choices when estimating parameter changes in pigs over time is in the study by Hidalgo et al. (2020). As an RRM with the complete dataset was taking months to run, these authors opted for estimating parameters using 3-yr data slices, each slice including up to three pig generations. Because REML was too expensive for a two-trait model (cubic cost with traits), Hidalgo et al. (2020) used the optimized Gibbs sampler Gibbs3f90, where computations increase approximately only linearly with the number of traits. Additionally, computations were reduced by pruning genotypes of young animals and pruning pedigrees to two generations of ancestors.

### Recommendations

Parameter estimation is likely to be relatively unbiased with any dataset in an unselected population, although using genomic information would reduce SEs of estimation. However, when selection present, it is prudent to use at least two to three generations of data. With GS, it is important to include the genomic information for animals with phenotypes, and genotypes from progeny without phenotypes can be removed to reduce computations. Models with few traits can be analyzed with REML especially if algorithms use efficient sparse matrix techniques for dense blocks. Models with large numbers of traits are best analyzed with a Gibbs sampler. When parameter changes are expected, the least expensive option is to estimate parameters using data slices, with pedigrees and genotypes truncated to limit computations.

## Methods for GWASs

### Current status

GWASs seek to map or find genes and genomic regions related to traits or diseases via interrogating genotype–phenotype relationships. Association studies emerged after QTL mapping in plants and animals and linkage mapping in humans. With the development of efficient genotyping technology covering the whole genome, GWAS started to populate after 2005. Currently, GWAS has been applied to study nearly all available traits across model and non-model organisms. As of August 5, 2020, the human GWAS Catalog contained 4,671 publications and 196,813 associations, and the Animal QTLdb had collected 2,308 publications and 191,422 associations across seven animal species (MacArthur et al., 2017; Hu et al., 2019; Tian et al., 2020). There were a total of 142,261 QTLs reported for cattle from 1,001 publications curated into the Animal QTLdb database. These QTLs involve 646 different traits that comprehensively characterize an animal regarding production, reproduction, health, body conformation, and efficiency.

GWAS has evolved in many ways in the past 20 yr. Sample size of GWAS has increased from hundreds in first-generation studies to hundreds of thousands such as the UK Biobank studies in humans (https://www.ukbiobank.ac.uk/) and studies involving industry-generated databases in livestock animals (Jiang et al., 2019b). More and more traits are being studied in GWAS, ranging from directly observable phenotypes, such as diseases, body type, and reproduction, to molecular or intermediate traits, such as levels of gene expression, methylation, proteins, metabolites, and even recombination rates. Recently, the availability of a comprehensive set of phenotypic and medical records in humans offered the possibility of phenome-wide association studies for better understanding genetic associations (Denny et al., 2010). Breeding values in livestock species have been routinely calculated based on hundreds or more relatives for selection purposes. These breeding values provide much more accurate phenotype data than individual animal phenotypes for GWAS, but the variation in reliability needs to be accounted for, especially in small samples (Garrick et al., 2009). Genotype data in GWAS have also improved with the development of genotyping and sequencing techniques. The earliest GWAS included thousands of SNPs, while some of the recent GWASs used whole-genome sequences. Nonetheless, imputation is always an economic way of increasing the number of variants and coverage of the genome.

### GWAS methods

All GWAS methods seek to model the relationship between genotype and phenotype. The classical quantitative genetics model assumes the genotype–phenotype relationship to be P = G + E, where P is phenotypic value, G is genotypic value, and E is environmental effect. Despite different levels of assumptions, GWAS models need to be close to this quantitative genetics model to be valid.

A single-marker test is the most popular method of GWAS. This method takes the form of a logistic regression for case–control studies of diseases and a linear regression for GWAS of quantitative traits. Historically, the single-marker test evolved from a simple regression to a regression with principal components to account for population structure (Price et al., 2006), then to a mixed model approach to account for sample relatedness (Yu et al., 2006). A mixed model with a single-marker test can be formulated as P = A + SNP_i_ + E, where A is a random animal effect or individual genotypic value and SNP_i_ is a fixed effect for a candidate SNP. The relatedness between samples can be modeled as a variance–covariance matrix of A via a genomic or pedigree relationship matrix (VanRaden, 2008). Compared with the classical quantitative genetics model, this mixed model replaced G with A + SNP_i_ and introduced some redundancy between A and SNP_i_. Because SNP_i_ is modeled as a fixed effect and A as random, the mixed model GWASs lose little power due to the overlap.

In addition to single-marker tests, GS models are often used in livestock GWAS (Fernando and Garrick, 2013; Misztal et al., 2014a; Aguilar et al., 2019). Instead of testing one marker at a time, GS models include all SNPs in the model, thus, $P=SNP_{1}+SNP_{2}+\ldots +SNP_{m}+E$, where SNP_i_ is the effect of SNP_i_, i = 1, …, m. Comparing this model and the classical quantitative genetics model, G is replaced with SNP_1_ + SNP_2_ + … + SNP_m_, assuming that genome-wide SNPs capture most of the QTL effects in the genome. Because this model is often solved by assuming random SNP effects or under a Bayesian framework, a frequentist statistical test or *P*-value is lacking, and SNP effect sizes or proportions of explained genetic variance are commonly used as evidence to support associations. Recently, Aguilar et al. (2019) developed a single-step GWAS (**SSGWAS**) approach to implement *P*-values for single-marker GWAS studies within the ssGWAS framework. Still, LD between SNPs may impact a full-model GWAS approach more than a single-marker mixed model test; thus, a full-model GWAS could be less powerful but more accurate than a single-marker test.

### Tips and quality assurance of GWAS

Like any other data analysis research, data quality is essential to ensure a valid GWAS. Quality checks are especially important for small-sample GWAS because data issues can easily lead to many more false-positive than true-positive results. Quality assurance procedures can be applied both before and after a GWAS analysis. We can check phenotype and genotype data before performing an association test. Generally, phenotypic values need to approximately follow a normal distribution, and outliers removed. We can check the quality of genotype data by checking Mendelian inheritance in the pedigree, perform a Hardy–Weinberg equilibrium test, and, depending on sample size, filter minor allele frequencies (**MAF**s) at 5% or 1% levels. Studies with small sample sizes need to use more stringent MAF cutoff levels to ensure that there are enough individuals carrying a minor allele in the sample (e.g., no less than 10).

After GWAS, we can still check the quality of the results using a quantile–quantile plot (**QQ** plot) or a Manhattan plot of *P*-values. In a QQ plot, we expect to see a uniform distribution of *P*-values by assuming that the majority of SNPs are not associated with the trait. In a Manhattan plot, we expect the top GWAS signals to be supported by clusters of SNPs in LD with the underlying causal variant(s). Those singled out significant SNPs in a Manhattan plot are likely due to data quality or analysis issues that sneaked through the quality control procedures.

### GWAS in dairy cattle and intersection with functional genomics data

There are some unique features in livestock GWAS, particularly in dairy cattle. Firstly, the livestock industry has generated large volumes of genotype and phenotype data for selective breeding. For example, the Council on Dairy Cattle Breeding (CDCB) in the United States maintains a dairy genomics database with millions of genotyped cattle and hundreds of millions of phenotypic records on a variety of economically important traits (https://www.uscdcb.com/). While GS has been successful with these data, they also provide uniquely powerful opportunities for GWAS and other genetics research. To date, this database has enabled many large-sample, powerful GWASs that revealed QTLs and genomic regions for many production, reproduction, health, and body conformation traits in dairy cattle (Cole et al., 2011; Jiang et al., 2017; Jiang et al., 2019a, 2019b). Secondly, a sizeable number of animals in dairy cattle populations have highly accurate breeding values estimated with data from all relatives that can have close to 100% reliability, particularly bulls with thousands of daughter records. By using a smaller number of dairy bulls with large numbers of progeny phenotypes, sequence-level GWAS and fine-mapping studies with millions of SNP variants become easily achievable (Jiang et al., 2019a).

After so many GWAS studies conducted and reported in the literature, a common question is what is next after GWAS? One answer is to go after causal variants. Although the dairy genomics database provides powerful data for dairy cattle GWAS, the high level of LD in the cattle genome makes the identification of causal variants difficult. The ongoing effort of the Functional Annotation of Animal Genomes (**FAANG**) (Giuffra and Tuggle, 2019) project will generate useful information to help find causal variants under GWAS peaks. However, there could be many years before we can easily identify causative variants after a GWAS.

An easier target after GWAS is to generate knowledge on where the causal/functional variants are in the genome. This knowledge can help fine-mapping studies and provide useful prior information for GS. We have conducted a few preliminary studies by integrating GWAS results and functional genomics datasets in cattle. Firstly, we explored transcriptome data across multiple tissues in cattle (Fang et al., 2020). Specifically, we identified tissue-specific expressed genes and tested whether GWAS signals are enriched in genes specifically expressed in some tissues. As a result, we detected relevant tissues for 45 dairy traits, including immune-related tissues for fertility, brain and neural tissues for milk production, and growth-related tissues for body conformation. Secondly, we explored methylome data in cattle sperm and found interesting intersections of sperm methylation data and GWAS results for male fertility traits (Fang et al., 2019). Lastly, we studied the intersection of histone marks and GWAS signals in cattle (Liu et al., 2020). By cross-mapping human epigenomics data to the cattle genome, we reported relevant tissues based on epigenome information for many dairy traits, including immune tissues for health and reproduction traits, multiple tissues for milk production and body conformation traits, and thyroid for genome differences between beef and dairy cattle. With more functional genomics data being generated by current FAANG efforts, more useful enrichment between GWAS results and functional genomic regions will be revealed that can improve the power of fine mapping and accuracy of GS.

### GWAS for complex models accounting for population structure with GBLUP and ssGBLUP

#### GBLUP and ssGBLUP

GBLUP is extensively used in the animal breeding industries for incorporating genomic information into the genetic evaluation of livestock species. Single-step GBLUP (Misztal et al., 2013) additionally accounts for ungenotyped individuals and has been adopted by many breed associations and private entities managing large-scale breeding programs. Although the main goal of GBLUP is the prediction of breeding values, a secondary interest focuses on performing GWASs.

Several methods have been proposed and successfully applied for embedding GWAS into genomic prediction models. Most of the methods commonly avoid formal hypothesis testing and resort to the estimation of SNP effects, relying on visual inspection of graphical outputs to determine candidate regions (Wang et al., 2012; Garcia et al., 2020). This approach requires visual inspection of Manhattan plots to determine association peaks. With the advent of high throughput phenomics and transcriptomics, a more formal testing approach with automatic discovery thresholds is more appealing for two reasons. First, it may not be feasible to inspect thousands of Manhattan plots and sort the position of each association peak, and second, when QTLs are mapped for large numbers of traits, it is necessary to correct for multiple testing.

With the widespread usage of ssGBLUP for genomic prediction across the industry, it is important that any GWA model incorporates information not only from genotyped individuals but also from their ungenotyped relatives that have been extensively phenotyped.

#### Genome-wide association tests from GBLUP and ssGBLUP

When all phenotyped animals are also genotyped, the animal-centric GBLUP model is represented by equation 4:

$$\begin{array}{l} y=X\beta +a+e \end{array}$$ (4)

where ***y*** is the vector of phenotypic values, ***β*** is the vector of fixed effects, ***X*** is the incidence matrix of fixed effects, ***a*** is the vector of breeding values, and e is the vector of residuals. Vectors ***a*** and e are assumed to follow a Gaussian distribution, thus $a\;∼\;N\;(0,G\sigma_{A}^{2}$) and $e\;∼\;N\;(0,I\sigma_{e}^{2})$. Matrix ***G*** is the genomic relationship matrix (VanRaden, 2008) that is computed using the matrix of standardized genotypes **Z** as **G**$\;=Z\;Z^{\prime }$.

Alternatively, an SNP-centric GBLUP (Meuwissen et al., 2001) model is presented in equation 5:

$$\begin{array}{l} y=X\beta +Zg+e \end{array}$$ (5)

where **y**, **X**, **b**, **Z**, and **e** are as defined above and $g\;∼\;N\;(0,I\sigma_{g}^{2}$) is the vector of random SNP effects, where $\sigma_{g}^{2}\;$is the variance of each SNP effect. The vector of breeding values ***a*** in equation 4 is equal to ***Zg*** in equation 5.

Strandén and Garrick (2009) showed that estimates of the SNP effects in equation 5 can be obtained by back-solving from the solutions to the vector of breeding values **a** in equation 4 using the expression $\hat{g}=Z^{\prime }G^{-1}\hat{a}$. Gualdrón Duarte et al. (2014) showed that instead of directly using ***g*** for performing genome-wide association, one could build a formal test statistic by computing the variance associated with estimated SNP effects (equation 6):

$$\begin{array}{l} var\left(\hat{g}\right)=Z\prime G^{-1}Z\sigma_{A}^{2}-Z{\;}^{\prime }\;G^{-1}C^{aa}G^{-1}Z \end{array}$$ (6)

where $C^{aa}$ is the portion of the mixed model equations associated with elements in vector **a** from equation 4, and then dividing the back-solved estimated SNP effects by their SEs (equation 7). For further details, see the section on Significance testing below:

$$\begin{array}{l} t_{i}={\hat{g}}_{i}/\sqrt{var({\hat{g}}_{i})} \end{array}$$ (7)

It is not necessary to compute the whole matrix product in equation 6, only the products involving diagonal elements. This can be easily parallelized even for a very large number of SNP. Also, $G^{-1}$ and $G^{-1}C^{aa}G^{-1}$ must be computed only once per trait.

Alternatively, consider augmenting the model in equation 4 by including a single fixed SNP effect, one at a time to test for association (equation 8):

$$\begin{array}{l} y=X\beta +a+z_{i}b_{i}+e \end{array}$$ (8)

where ***z***_***i***_ is the column of the standardized genotype matrix Z corresponding to the ith SNP, and *b*_*i*_ is the fixed effect of the allelic dosage of the ith SNP on the phenotype, and all the other quantities are as described for equation 4.

Instead of refitting equation 8 for every fixed SNP effect, the Efficient Mixed-Model Association eXpedited (**EMMAX**) procedure (Kang et al., 2010) involves first fitting the null model (equation 4) and estimating its variance components, and then solving the mixed model equations of equation 8 using the variance ratios estimated from equation 4. Finally, a test of association is obtained by dividing the estimated SNP effects in equation 8 by their SEs (equation 9):

$$\begin{array}{l} t_{i}={\hat{b}}_{i}/\sqrt{var({\hat{b}}_{i})} \end{array}$$ (9)

Bernal Rubio et al. (2016) published an analytical proof that shows the mathematical equivalence between equations 7 and 9. Specifically:

$$\begin{array}{l} {\hat{g}}_{i}=\frac{\sigma_{u}^{2}}{var(b_{i})}{\hat{b}}_{i} \end{array}$$ (10)

and

$$\begin{array}{l} var({\hat{g}}_{i})=\frac{{\left(\sigma_{u}^{2}\right)}^{2}}{var({\hat{b}}_{i})} \end{array}$$ (11)

This is an important result because it shows that all the statistical properties of EMMAX (Kang et al., 2010; Teyssèdre et al., 2012; Legarra et al., 2018) apply to the test of association derived from back-transformation of breeding values (equation 7). Computing the test via back-transformation can be done in a computationally efficient way without having to recompute the mixed model equations for each SNP replacement.

#### Genome-wide association for ssGBLUP

The calculation of $var\left(\hat{g}\right)$ for GBLUP was extended to ssGBLUP permitting the construction of a formal test statistic by computing the variance associated with estimated SNP effects (Aguilar et al., 2019; Lourenco et al., 2020). Let the vector of breeding values ***a*** in equation 3 be partitioned into two subvectors, $a=\left[\frac{a_{1}}{a_{2}}\right]$, where $a_{2}$ corresponds to the subvector of genotyped animals and $a_{1}$ corresponds to the subvector of non-genotyped animals. Then, the estimation algorithm proceeds as follows:

1. Create the ssGBLUP equations
2. Obtain the sparse inverse matrix of the left-hand side of the mixed model equations **C**.
3. Extract the submatrix corresponding to the genotyped animals, $C^{a_{2}a_{2}}$, from matrix C. This submatrix contains the prediction error (co)variances of the estimated breeding values of the genotyped animals, ${\hat{a}}_{2}$, i.e., $Var\left(a-{\hat{a}}_{2}\right)=C^{a_{2}a_{2}}$.
4. Backsolve for estimates of SNP effects as follows: $\hat{g}|\hat{u}=\frac{1}{2\Sigma p_{i}q_{i}}G^{-1}\;\;\;{\hat{a}}_{2}$
5. Obtain individual prediction error variances of SNP effects using the expression from equation 6:

$$\begin{array}{l} Var\left({\hat{g}}_{i}\right)=\frac{1}{2\overset{}{\sum p_{i}q_{i}}}z_{i}^{\prime }G^{-1}\left(G\sigma_{a}^{2}-C^{a_{2}a_{2}}\right)G^{-1}z_{i}\frac{1}{2\overset{}{\sum p_{i}q_{i}}} \end{array}$$

where $z_{i}$ is the ith column of matrix Z, corresponding to genotypes of marker i across individuals:

6. Calculate the test statistic from equation 7:

$$\begin{array}{l} t_{i}={\hat{g}}_{i}/\sqrt{var({\hat{g}}_{i}}) \end{array}$$

### Significance testing

Under the null hypothesis of no association, the test statistic in equations 7 or 9 follows a standard Gaussian distribution, thus an associated two-tailed *P*-value for the test is computed with equation 12:

$$\begin{array}{l} p_{i}\;=\;2(1-{\Phi \;}^{-1}\;(|ti|) \end{array}$$ (12)

where ${\;\Phi \;}^{-1}$ is the inverse cumulative density function of the standard Gaussian distribution.

Gualdrón Duarte et al. (2014) performed stochastic plasmode simulations and empirically confirmed the null distribution of the test statistic. Wang et al. (2020) compared the distribution of this test statistic to alternative tests and showed that, for relatively low marker densities, where some markers explain a large proportion of the phenotypic variance, this test is suboptimal and loses power because the markers in question are used in ***G*** of equation 4. Moreover, these authors propose corrections for this test statistic to regain some power. Conversely, these authors showed that for the typical situation of dense markers, where each marker explains a small proportion of the phenotypic variance, this test statistic performs well and there is no need to apply corrections.

Once a set of association *P*-values for each SNP is computed, a multiple test correction is recommended to establish a significance threshold. Gualdrón Duarte et al. (2014) showed with plasmode simulations that establishing a Bonferroni threshold for a highly colinear SNP set results in overly conservative thresholds. Because the extent of LD in livestock species if much larger than in humans (Qanbari, 2020), a Bonferroni threshold is probably not recommended for GWASs in animal science. An empirical permutation Bonferroni threshold is likely better, but it is computationally demanding, and it must be applied separately for each phenotypic trait. A Bonferroni correction usually controls the type-one error rate at the experiment-wise level but at the expense of a dramatic decrease in power. In these circumstances, a less stringent, yet robust multiple test correction procedure such as estimating the proportion of false positives (Fernando et al., 2004) offers a good tradeoff between controlling false positives and false negatives. Fernando et al. (2004) demonstrated that the proportion of false positives controls type-one error rate even for correlated tests and that it can be applied to many of the traits simultaneously (as it happens in transcriptomics analyses) to control the proportion of false positives for the whole experiment.

### Confidence intervals for the position of a QTL peak

Once a significant association peak is detected using the test in equation 7, a confidence interval can be computed by using a jackknife procedure (Hayes, 2013). In brief, the dataset is split into two parts by randomly assigning each observation to one of the two sets. A genome-wide association is performed for each data partition and the position of the association peak in each partition is recorded. The procedure is repeated K times and the standard deviation of the association peak is computed for each data partition using equation 13:

$$\begin{array}{l} se\left(\bar{x}\right)=\sqrt{\frac{1}{4K}\underset{k=1}{\overset{K}{\sum }}\;{\left(v_{1k}-v_{2k}\right)}^{2}} \end{array}$$ (13)

where $v_{1k}$ and $v_{2k}$ are the positions of the association peaks for data partitions 1 and 2 in iteration k. Lastly, a 95% confidence interval for the position of the peak is obtained with equation 14:

$$\begin{array}{l} v\;\pm z_{97.5}se(\bar{x}) \end{array}$$ (14)

where v is the position of the association peak estimated using the full dataset, and $z_{97.5}$ = 1.96 is the value of the 97.5 percentile of the standard Gaussian distribution. The described procedure does not have to be applied genome-wide, but locally, including only a set of SNPs around the association peak. This procedure can be used to find plausible genomic intervals for the association peak, which are particularly useful for post hoc bioinformatic analyses, such as searching for genes and other annotated genomic features (Casiró et al., 2017; Velez-Irizarry et al., 2019).

### Estimation of proportion of variance explained by a QTL tagged by an association peak

The variance explained by a putative QTL is an important genetic parameter that is often reported in association studies. The variance associated with a QTL peak can be computed in two ways with the procedure described above. The most common way consists of selecting a window around the association peak. This can be done, for instance, using the confidence interval method presented above or by selecting an arbitrary interval (Gualdrón Duarte et al., 2016) or by inspecting the Manhattan plot and searching for all the SNPs that are significantly associated with the trait (Bernal Rubio et al., 2015). Subsequently, two relationship matrices are computed: ***G***_***w***_ based on the SNP included in the selected window and ***G***_*-w*_ based on all the other SNPs. Then, a linear mixed model is fit including two random effects, one associated with each relationship matrix (equation 15):

$$\begin{array}{l} y=X\beta +a_{-w}+a_{w}+e \end{array}$$ (15)

where $a_{-w}\;∼N\;\;(0,G_{-w}\sigma_{-w}^{2}$) and $a_{w}∼\;N\;(0,G_{w}\sigma_{w}^{2}$). Lastly, the proportion of the variance explained by the association peak is obtained using equation 16:

$$\begin{array}{l} h_{w}^{2}=\frac{{\hat{\sigma }}_{w}^{2}}{{\hat{\sigma }}_{-w}^{2}+{\hat{\sigma }}_{w}^{2}+{\hat{\sigma }}_{e}^{2}}. \end{array}$$ (16)

There are two computational bottlenecks when using this procedure. The first one is the recomputation of the two relationship matrices, especially the computation of $G_{-w}$. The second one is solving the mixed model equations. Computation of $G_{w}$ may also be computationally onerous, depending on the number of animals, but this matrix is generally much smaller than $G_{-w}$ because it includes only a few SNPs (in the order of tens or hundreds of SNPs vs. tens of thousands).

A computationally less onerous approximation (Casiró et al., 2017) consists of resorting to equation 8, where the SNP is assumed to be a fixed effect, and then the variance explained by the ith fixed SNP effect is approximated using equation 17:

$$\begin{array}{l} {\hat{\sigma }}_{i}^{2}=var(z_{i}){\hat{b}}_{i}^{2} \end{array}$$ (17)

where $var(z_{i})$ is the variance of the ith column of the ***Z*** matrix and ${\hat{b}}_{i}$ can be obtained using equation 8. It is important to note that all these procedures only produce gross approximations to the explained variance, and they will likely overestimate the variance explained by the QTL represented by the association peak, but these expressions may be useful to prioritize QTLs according to the relative magnitude of their effect sizes.

## Increased Fluctuations of Genetic Evaluations with GS

GS introduced considerable changes in the field of animal breeding and genetics since its first implementation in 2009. The most important change was the increase in genetic gain resulting from the decrease in generation interval and greater accuracy of prediction of GEBV. However, the widespread adoption of GS brought some challenges, namely high computational demands and the need for new methods. Additionally, reports have emerged on differences in variance components (Hidalgo et al., 2020) and inbreeding levels (Makanjuola et al., 2020) when genomic information is used. Those differences do not necessarily imply that genomic information is causing a reduction in additive genetic variance or an increase in inbreeding. It means that the use of a new source of information and recent methods results in contrasting values to those from pedigree-based approaches. Once selection is based on genomic information, it is fair to use this information in all steps of the selection process to avoid biases.

Additional changes that have been reported since GS started being used relate to GEBV. Fluctuations in GEBV in subsequent evaluations have been observed even for animals that have no phenotypes added to the evaluation. Questions on the reason for such fluctuations are frequent in the livestock industry. This is because, under traditional BLUP, animals without new data in subsequent evaluations had stable EBV even when their accuracy was low. However, changes in EBV could still be observed for some animals when fixed effects were redefined, independently of accuracy levels. Provided that no model changes are made, EBV changes are limited to animals that have new data and those directly related to them through the pedigree. In fact, under BLUP, the EBV of an animal is conditioned only on the breeding value of its sire and dam. As pedigree links are sparse, EBV can be considered stable because most of the animals will have no changes. The high stability of EBV from BLUP generated great confidence in the method. Additionally, stability is an important factor because bulls are priced according to their genetic merit, and large, unexpected changes in EBV would create oscillations in the semen market.

Pedigree relationships in BLUP are only expectations of the proportion of alleles shared among individuals, whereas in GBLUP or ssGBLUP the genomic information is used to better capture relationships at the gene level. Relationships based on SNPs are termed realized relationships, being, therefore, based on the observed proportion of shared alleles that are identical by state. Because of that, all genotyped animals can have some level of relationship even though they do not share common ancestors in the current population.

Although the pedigree relationship matrix (**A**) is sparse, the genomic relationship matrix (**G**) is dense, which means animals are more connected. Because of the stronger connections, when phenotypes are added to a portion of the genotyped animals in the evaluation, changes in GEBV are more frequent because this information is shared among nearly all genotyped animals. When only a portion of the pedigree animals is genotyped, ssGBLUP (Aguilar et al., 2010; Christensen and Lund, 2010) is the method of choice because it combines pedigree and genomic relationships into a realized relationship matrix (**H**). Another feature of ssGBLUP is that genomic information is back propagated (i.e., implicitly imputed) to non-genotyped animals that are related to genotyped animals through the pedigree (Legarra et al., 2009). This means relationships among non-genotyped animals are enhanced by the genomic information of their relatives, which can create additional fluctuations in GEBV for non-genotyped animals.

**Figure 1.**
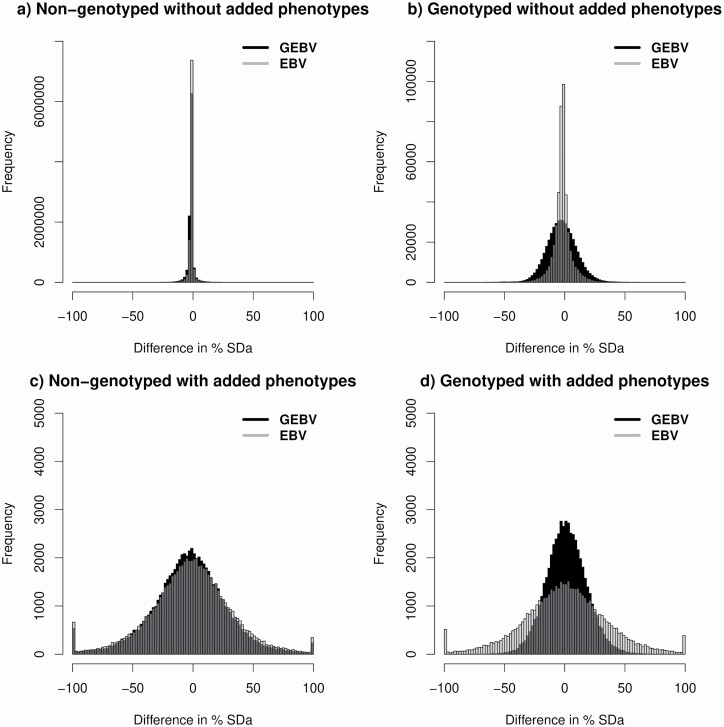
Distribution of EBV from BLUP and GEBV from ssGBLUP for (a) non-genotyped animals without added phenotypes, (b) genotyped animals without added phenotypes, (c) non-genotyped animals with added phenotypes, and (d) genotyped animals with added phenotypes. Changes were expressed as a percentage of the SDa of 27.01.

To investigate changes in EBV and GEBV when phenotypes for some animals are added to the evaluation, we mimicked an evaluation system with two yearly runs using an American Angus dataset. The first evaluation used 4.26 M phenotypes for postweaning gain until July 2017 (Jul2017) and the second evaluation used 4.38 M phenotypes until December 2017 (Dec2017). The number of animals in the pedigree and with genotypes was kept constant between the two subsequent evaluations at 10,661,517 and 509,072, respectively. A total of 54,798 genotyped and 69,996 non-genotyped animals had phenotypes added to the Dec2017 evaluation. For each evaluation, EBV and GEBV were computed by BLUP and ssGBLUP, respectively, using the BLUPF90 software suite (Misztal et al., 2014b). Figure 1 shows the distribution of changes in EBV and GEBV from Jul2017 to Dec2017 for genotyped and non-genotyped animals with and without added phenotypes. Negative values mean a decrease in breeding values from Jul2017 to Dec2017.

Changes in BLUP EBV and ssGBLUP GEBV for non-genotyped animals that had no data added from Jul2017 to Dec2017 (Figure 1a) were minimal, averaging 1.5% and 1.8% of one additive genetic standard deviation (**SDa**), respectively. The small differences between the distribution of EBV and GEBV can be attributed to the contributions of genotyped relatives to non-genotyped animals (i.e., enhanced relationships). When looking at genotyped animals without added phenotypes in subsequent evaluations (Figure 1b), changes in EBV and GEBV had a wider distribution but EBV changes had a higher frequency of very small differences (i.e., close to zero), whereas GEBV changed more for a larger number of animals. These are the most concerning changes to breeders because new data were not added to those animals; however, they are genotyped and are more related through **H**, which means they share more alleles with animals that had additional phenotypes in the Dec2017 evaluation. Although these changes are surprising to breeders, they are expected based on GS theory. The average change in GEBV for genotyped animals without added phenotypes was 2.4% of 1 SDa and in EBV was 2.0%; however, changes were more extreme for EBV (up to 2 SDa) than for GEBV (up to 0.8 SDa). The distribution of changes for EBV and GEBV for non-genotyped animals with added phenotypes was very similar (Figure 1c), with a slightly higher frequency of extreme changes for EBV. For genotyped animals with phenotypes added from July2017 to Dec2017 (Figure 1d), many more animals had smaller changes in GEBV than in EBV. The maximum change was 2.8 SDa for EBV and 0.9 SDa for GEBV. 

**Figure 2.**
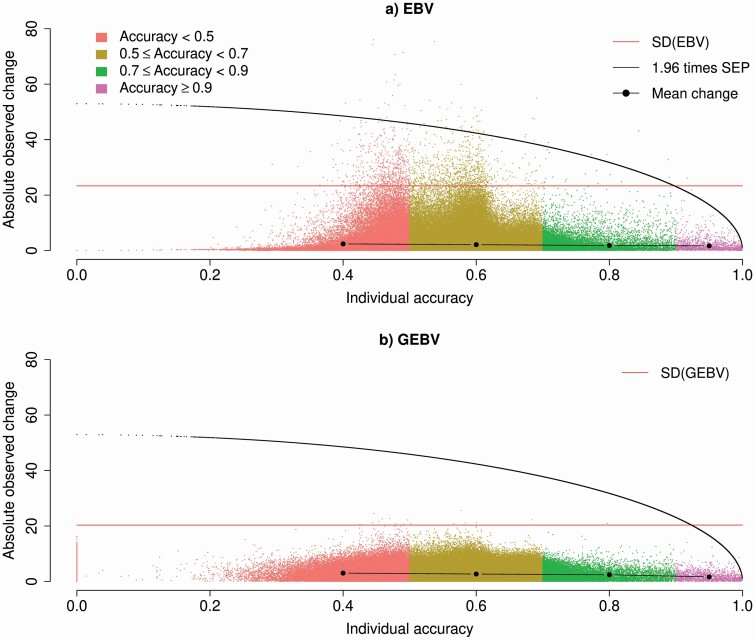
Absolute changes in EBV and GEBV as a function of individual accuracies. Evaluations were based on July 2017 and December 2017 data, and accuracies were based on the SEP from BLUP (i.e., pedigree and phenotypes). Different classes of individual accuracies are based on information from pedigree and phenotypes only.

If the question is why more extreme changes are observed for EBV than for GEBV, the answer is simple; although average changes are higher for GEBV, the changes in (G)EBV are bounded by the accuracy of (G)EBV. As more information is used to compute GEBV than EBV, their accuracy is higher, and consequently, their possible changes are less extreme. When changes from Jul2017 to Dec2017 for all genotyped animals were investigated as a function of individual accuracy and SE of prediction, we observed more changes in EBV and GEBV for animals with lower accuracy (Figure 2).

Many animals had changes in EBV that were over 1.96 times the standard error of prediction (**SEP**) (Figure 2a). The 1.96 × SEP corresponds to the 95% confidence interval of possible changes for EBV. When the absolute changes in GEBV were plotted against the possible changes (Figure 2b), values were much smaller than the possible changes, and very few animals had changes over 1 SD of GEBV. Although more animals have general changes in GEBV than in EBV when additional phenotypes are added to the evaluation system, these changes are of lower magnitude.

In the context of genomic evaluation and assuming no new phenotypes are added, any change in the genomic relationship matrix can cause changes in GEBV. Misztal et al. (2020) showed that when the core animals used to construct the inverse of **G** in the algorithm for proven and young (Misztal et al., 2014c) are updated, the average change in GEBV is 5% of 1 SDa. Although the correlation between GEBV using different core groups is greater than 0.99, the maximum change can be as high as 1 SDa. The same authors also showed that modifications in the proportion of the pedigree relationship matrix that is blended to **G** to avoid singularity problems can cause considerable changes in GEBV.

Although the changes discussed here are from the methods based on genomic relationships, GEBVs from SNP effect methods are similarly subject to changes when new phenotypes are added to the evaluation system or when the training population is updated. The inclusion of new genotyped animals also causes changes in both methods.

Before GS was implemented, changes in EBV were mainly due to model changes, redefinition of contemporary groups, new variance components, and new data. In the latter, fluctuations are more substantial for animals with added phenotypes and their relatives through **A**, which is usually a limited group of animals. Although the average change in EBV is low, there are more extreme fluctuations than with GEBV; thus, changes in EBV for low accuracy animals are artificially low. Changes in GEBV are caused by the same factors as for EBV; however, changes occur for larger numbers of animals because all genotyped animals are somehow related through **G**. Although GEBVs have higher average changes, generally, changes are less extreme, a reflection of the higher accuracy of GEBV.

Change in GEBV for more animals when new phenotypic data are added is an inherent factor of the genomic evaluation system simply because genomic information connects more animals. One way to minimize the impact of these changes is to market groups of sires (e.g., semen from groups of sires) with high average accuracy instead of individual sires. Another way is to recognize that these changes are based on the GS theory and are part of the system.

## Conclusions

Although GS has been widely successful, many remaining issues are still being identified and addressed. More theoretical studies following analyses of large field datasets are needed to fully understand the effects of long-term GS. Parameter estimation with genomic information requires careful selection of data to minimize computations and biases. Methods for GWAS need to include strong LD and effects of inadequate modeling, especially with small datasets. Fluctuations of genomic predictions reflect limited prediction accuracies and can be managed to reduce risk and achieve high genetic gains.

## Abbreviations

A × A: additive by additive
BLUP: best linear unbiased prediction
CDCB: Council on Dairy Cattle Breeding
D × D: dominance by dominance
EMMAX: Efficient Mixed-Model Association eXpedited
FAANG: functional annotation of animal breeding
GBLUP: genomic best linear unbiased prediction
GEBV: genomic estimated breeding value
GREML: restricted maximum likelihood with genotyped animals
GS: genomic selection
GWAS: genome-wide association study
LD: linkage disequilibrium
LE: linkage equilibrium
MAF: minor allele frequency
QQ plot: quantile–quantile plot
QTL: quantitative trait loci
REML: restricted maximum likelihood
RRM: random regression model
SDa: additive genetic standard deviation
SEP: standard error of prediction
SNP: single-nucleotide polymorphism
ssGBLUP: single-step GBLUP
ssGREML: single-step restricted maximum likelihood with genotyped and ungenotyped animals
SSGWAS: single-step GWAS
